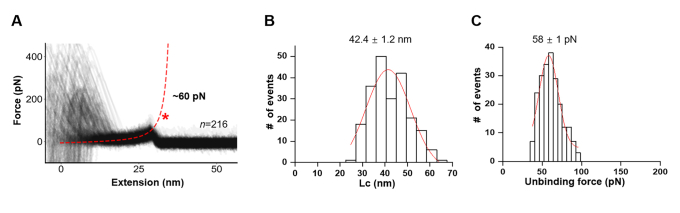# Mechanistic insights into the C-type lectin receptor CLEC12A-mediated immune recognition of monosodium urate crystal

**DOI:** 10.1016/j.jbc.2024.105765

**Published:** 2024-02-16

**Authors:** Hua Tang, Yuelong Xiao, Lei Qian, Zibin Wang, Ming Lu, Nan Yao, Ting Zhou, Fang Tian, Longxing Cao, Peng Zheng, Xianchi Dong

**Affiliations:** 1 State Key Laboratory of Pharmaceutical Biotechnology, Department of Biochemistry, School of Life Sciences, Nanjing University, Nanjing, China; 2 Engineering Research Center of Protein and Peptide Medicine, Ministry of Education, Nanjing, China; 3 State Key Laboratory of Coordination Chemistry, Chemistry and Biomedicine Innovation Center (ChemBIC), School of Chemistry and Chemical Engineering, Nanjing University, Nanjing, Jiangsu, China; 4 Westlake laboratory, Westlake University, Hangzhou, Zhejiang, China; 5 Institute of Artificial Intelligence Biomedicine, Nanjing University, Nanjing, China

**Keywords:** C-type lectin receptor, CLEC12A, monosodium urate crystal, single-molecule force spectroscopy, crystal structure, damage-associated molecular pattern, oligomerization, phagocytic vesicle

## Abstract

CLEC12A, a member of the C-type lectin receptor family involved in immune homeostasis, recognizes MSU crystals released from dying cells. However, the molecular mechanism underlying the CLEC12A-mediated recognition of MSU crystals remains unclear. Herein, we reported the crystal structure of the human CLEC12A-C-type lectin-like domain (CTLD) and identified a unique “basic patch” site on CLEC12A-CTLD that is necessary for the binding of MSU crystals. Meanwhile, we determined the interaction strength between CLEC12A-CTLD and MSU crystals using single-molecule force spectroscopy. Furthermore, we found that CLEC12A clusters at the cell membrane and seems to serve as an internalizing receptor of MSU crystals. Altogether, these findings provide mechanistic insights for understanding the molecular mechanisms underlying the interplay between CLEC12A and MSU crystals.

C-type lectin receptors (CLRs) are a large family of more than 1000 proteins that possess one or more C-type lectin-like domains (CTLDs). CLRs recognize a diverse range of endogenous (self) and exogenous (non-self) ligands through CTLDs in a Ca^2+^-dependent or Ca^2+^-independent manner, which is essential for the maintenance of immune homeostasis (1). The Dectin-1 cluster is a subgroup of CLRs, consisting of seven structurally related receptors including CLEC12A, CLEC-2, CLEC12B, CLEC9A, CLEC-1, Dectin-1, and LOX-1, which are involved in regulation of inflammation, infection, and other autoimmune diseases (2). CLEC12A (also known as MICL, CLL-1, KLRL1, and DCAL-2) is extensively expressed in myeloid cells, including monocytes, macrophages, granulocytes, and dendritic cells (3, 4). CLEC12A belongs to the type Ⅱ transmembrane receptor, consisting of an extracellular CTLD, a stalk region, a transmembrane region, and a cytoplasmic tail that harbors an ITIM (5). Upon ITIM phosphorylation by Src kinases SHP-1 and SHP-2, CLEC12A becomes tyrosine phosphorylated, triggering the inhibition of cellular activation (6, 7). CLEC12A-deficient mice show exacerbated inflammation in collagen antibody-induced arthritis (8) and reduction of IFN-I responses during lymphocytic choriomeningitis virus infection (9), indicating that CLEC12A plays important roles in negative regulatory of inflammation and positive antiviral responses.

It has been reported that CLEC12A is involved in the recognition of multiple kinds of molecular patterns. CLEC12A acts as an innate sensor of plasmodial hemozoin and contributes to the development of cerebral malaria (10). CLEC12A is also found to be involved in antigen uptake and cross-presentation by human dendritic cells, resulting in strong activation of antigen-activate T cells (11). Additionally, CLEC12A emerges as a promising therapeutic target for the treatment of AML and MDS (12, 13). Also, CLEC12A is a biomarker of AML cells and increasingly acts as CAR-T cell therapy target for AML patients (14). MSU crystal deposition in joints is the primary cause of the development of gout, and the gout flare exhibits an acute inflammatory response to deposited MSU crystals (15, 16). CLEC12A is identified as not only an innate sensor of plasmodial hemozoin but also a well-identified specific receptor for the recognition of MSU crystals from dead cells (17). CLEC12A senses MSU crystals and inhibits proinflammatory pathways by counteracting the activating Syk through ITIM motif (6, 7). Furthermore, MSU crystal detection by CLEC12A promotes cytosolic RNA-induced IFN-I production (9). Currently, the molecular mechanism of CLEC12A-mediated recognition of MSU crystals remains poorly understood.

In the present study, we report the crystal structure of human CLEC12A-CTLD, and identify the CLEC12A receptor directly binds to MSU crystals through a specific “basic patch”. Then, we further elucidate the binding force of CLEC12A receptor to MSU crystals at the single molecular level by utilizing AFM-SMFS method. In macrophages, we also find that CLEC12A mediates the recognition of MSU crystals in a manner of clustering upon sensing of MSU crystals and CLEC12A appears to serve as an endocytic receptor for MSU crystals. These results reveal molecular mechanisms underlying the CLEC12A-mediated recognition and uptake of MSU crystals, as well as provide mechanistic insights into understanding the interaction between CLEC12A and MSU crystals.

## Results

### Crystal structure of the hCLEC12A-C-type lectin-like domain

**Figure 1:**
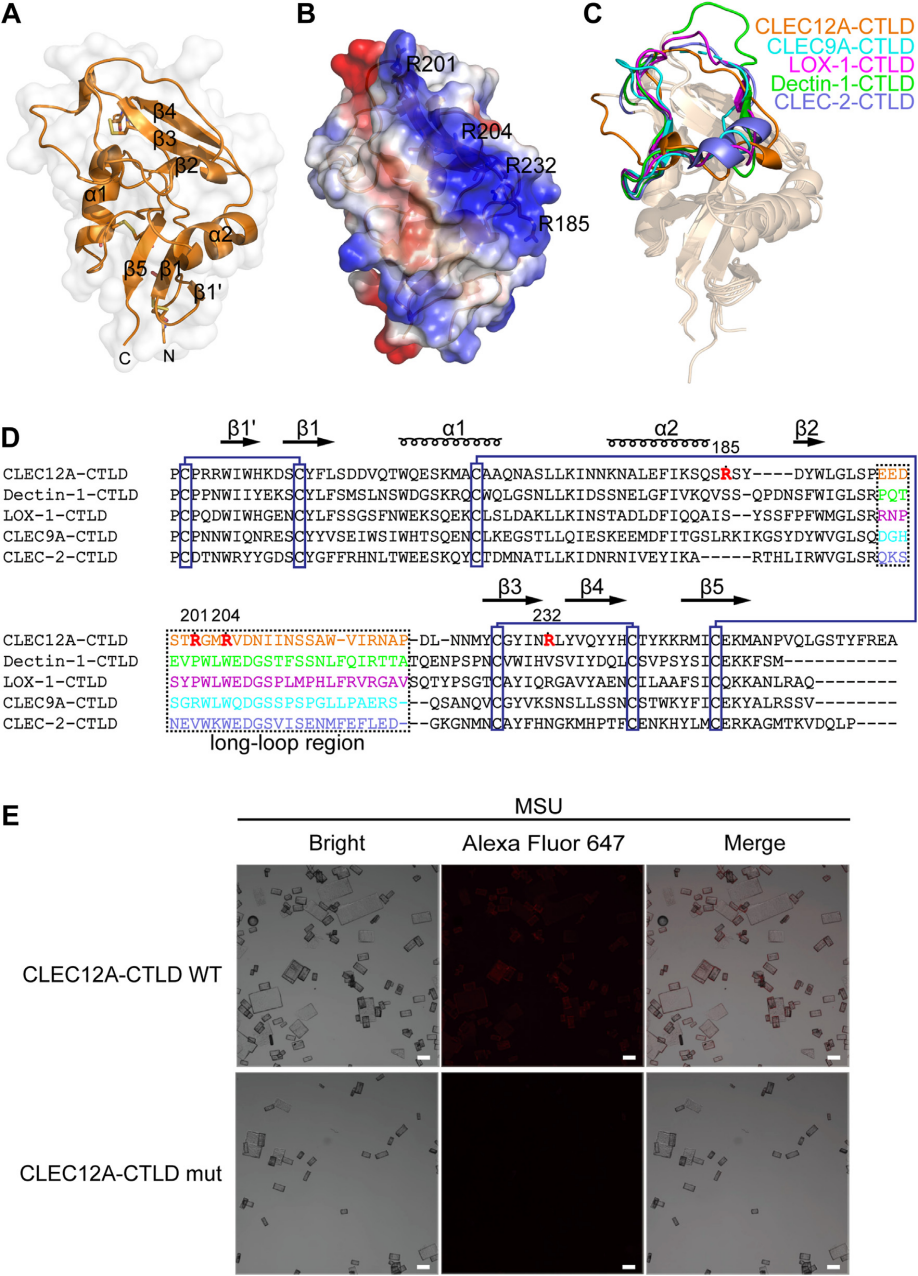
**CLEC12A directly binds to MSU crystals through a unique “basic patch” region.***A*, crystal structure of the hCLEC12A-CTLD. *B*, electrostatic potential surface of the hCLEC12A-CTLD structure. Positively-charged arginines positioned on “basic patch” are labelled. The *blue*, *white* and *red colors* correspond to net positive, neutral and negative charges, respectively. *C*, structural alignment of CTLDs among “Dectin-1” cluster. The long-loop regions from “Dectin-1” cluster are labelled and colored. Crystal structures of the CTLDs of hCLEC12A (*orange*), hCLEC9A (*cyan*, PDB:3VPP), hLOX-1 (*magenta*, PDB:1YPQ), mDectin-1 (*green*, PDB:2BPD) and hCLEC-2 (*slate*, PDB:2C6U) were shown as ribbon diagrams. *D*, sequence alignment of CTLDs among “Dectin-1” cluster. The loop regions are labeled by *rectangle*. Positively charged arginines at the “basic” patch are indicated in *red*. Six cysteine residues that are canonical in CTLDs are marked, indicating disulphide bond formation. *E*, confocal microscopy analysis and representative images of MSU crystals binding to wild-type CLEC12A-CTLD proteins labeled with Alexa Fluor 647, instead of mutant CLEC12A-CTLD proteins labeled with Alexa Fluor 647. Scale bar, 20 μm. CTLD, C-type lectin-like domain.

The human CLEC12A-CTLD protein (residues 132–255) was expressed in insect cells and purified by Ni-NTA affinity chromatography and SEC. The purified CLEC12A-CTLD protein was crystallized and diffracted to 2.58 Å, and the structure of CLEC12A-CTLD was solved by molecular replacement using the CTLD fragment of the CLEC9A as a search model. The structure of hCLEC12A-CTLD exhibits canonical features of the CTLD including two antiparallel β-sheets flanked by two α-helices (Fig. 1*A*), displaying a similar overall structure to the CTLDs from “Dectin-1” cluster members (18, 19, 20, 21). The “Dectin-1” cluster members possess similar CTLDs that are responsible for recognizing different ligands (22, 23). CLEC9A (also known as DNGR-1) binds to F-actin released by dying cells (24). CLEC-2 binds to the endogenous ligand podoplanin (25). LOX-1 senses oxidized low-density lipoprotein and heat shock protein (26); Dectin-1 (also known as CLEC7A) which recognizes β-glucans in fungi (27). Structural alignment of these structures reveal that these molecules share the similar overall conformation, and the root mean square deviations of the Cα atoms among CLEC12A, CLEC9A, CLEC-2, LOX-1, and Dectin-1 is between 0.8 Å to 1.2 Å, showing high overall structural similarity of CTLDs among “Dectin-1” cluster members (Fig. 1*C*). Sequence alignment and structural alignment show that distinct characteristics of these CTLDs are particularly focused on the long-loop regions that are distal from each other (Fig. 1, *C* and *D*), which suggests the diversity and specificity in ligand recognition by C-type lectin receptors (19). In the crystal structure of CLEC12A-CTLD, a long-loop region corresponding to residues from Glu196 to Asp221 on the CLEC12A-CTLD is distal from other CTLDs among “Dectin-1” cluster molecules (Fig. 1, *C* and *D*), implying that this long-loop region could be specifically responsible for ligand-binding.

### CLEC12A recognizes MSU crystals through a “basic patch”

**Supporting Figure S1:**
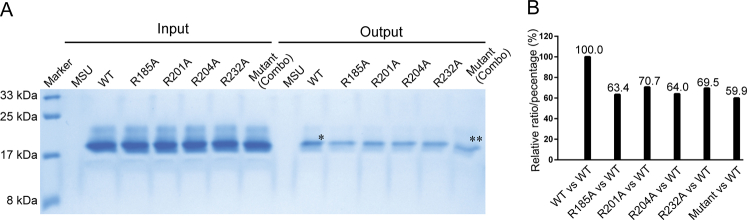


**Supporting Figure S2:**
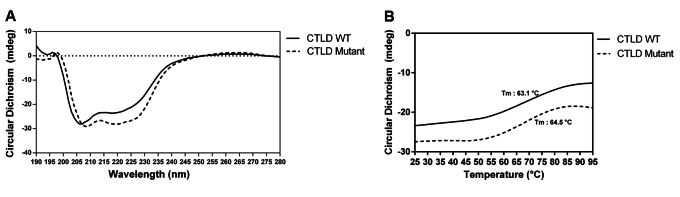


In the crystal structure of CLEC12A-CTLD, a “basic patch” region could be clearly observed, which is conformationally formed by positively-charged residues of Arg185, Arg201, Arg204, and Arg232 (Fig. 1*B*). We hypothesized that the “basic patch” on the CLEC12A-CTLD is required for the recognition of MSU crystals. Therefore, we generated four single mutants (R185A, R201A, R204A, or R232A on wild-type protein) and one quadruple mutant (CLEC12A-CTLD mutant), which included four mutations in a single protein: R185A, R201A, R204A, and R232A on wild-type CLEC12A-CTLD protein. Using these five mutants and wild-type of CLEC12A-CTLD proteins, we conducted protein-MSU crystal pulldown assay to figure out which residue is critical for the MSU binding. Pulldown result reveals that the quadruple mutant protein evidently disables the strong binding to MSU crystal (Fig. S1). In the meantime, both wild-type and CLEC12A-CTLD mutant proteins were used to perform CD spectrometry, which unveils that both wild-type and CLEC12A-CTLD mutant proteins feature similarly and fold correctly (Fig. S2). Furthermore, both wild-type and CLEC12A-CTLD mutant proteins were labelled with the Alexa Fluor 647, and used for MSU crystals binding assay. Alexa Fluor 647-fluorescence of the crystals was detected by fluorescence microscopy. The wild-type CLEC12A-CTLD exhibits obvious adsorption on MSU crystals by confocal microscopy, while the CLEC12A-CTLD mutant showed barely adsorption on MSU crystals (Fig. 1*E*). These results suggest that the unique “basic patch” on the CLEC12A-CTLD is essential for the recognition and binding of MSU crystals. The negatively-charged surfaces on the MSU crystals are preferentially recognized by other cell surface receptors (28, 29, 30), which is also adopted by CLEC12A.

### Single-molecule force spectroscopy determines a strong interaction force between CLEC12A-CTLD and MSU crystals

**Figure 2:**
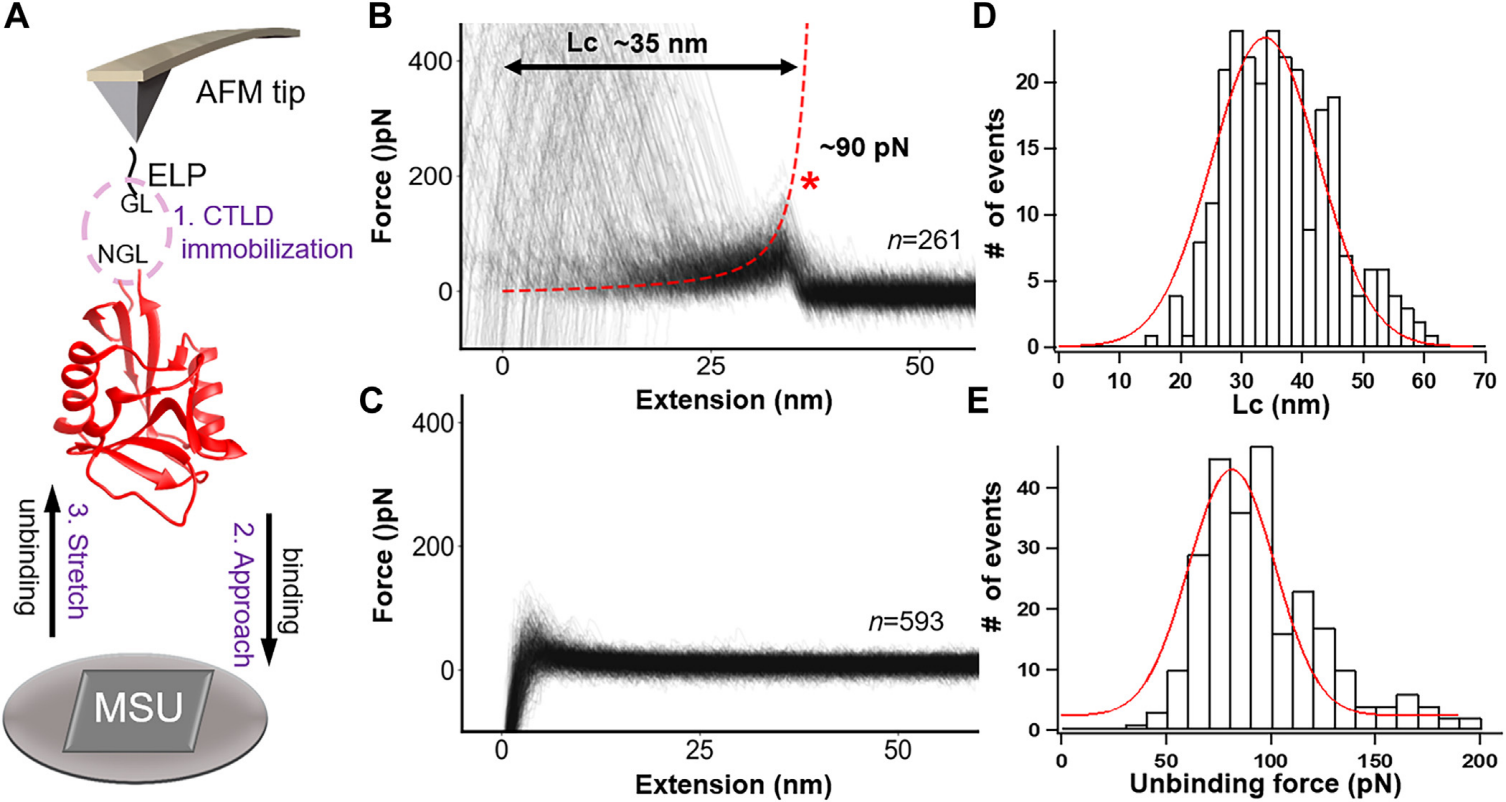
**Measurement of the binding strength between hCLEC12A-CTLD and MSU crystal by AFM-SMFS.***A*, schematics of the AFM-SMFS set-up for the measurement of the binding strength between hCLEC12A-CTLD and MSU crystal. *B*, force-extension curves of the unbinding between CLEC12A-CTLD and MSU crystal, showing a force peak with a contour length of ∼35 nm and a force of ∼90 pN. *C*, a control experiment using only ELP showed no binding event with MSU crystal. *D*, the histogram of contour length (Lc) of the unbinding force peak showed an average length of 35 nm. *E*, the histogram of unbinding force between CLEC12A-CTLD and MSU crystal showed an average force of 90 pN. CTLD, C-type lectin-like domain.

To directly determine the molecular interaction force between hCLEC12A-CTLD and MSU crystals, we designed and performed AFM-SMFS experiment (Fig. 2*A*). AFM-SMFS is a powerful tool to study protein folding or unfolding and receptor-ligand interaction in biological systems due to its operability in physiological conditions, rapid sample preparation, and versatile molecular manipulations (31). In the experiment group with CLEC12A-CTLD immobilized on the AFM tip, significant force-extension curves with a clear force peak were observed (Fig. 2*B*, n = 261). Also, a control experiment group without immobilized CLEC12A-CTLD on the AFM tip was performed, showing no obvious force peak (Fig. 2*C*, n = 593). Therefore, the unbinding force peak was indeed from the interaction between CLEC12A-CTLD and the MSU crystals. In addition, the force-extension curve can be well fitted by the worm-like chain model that is usually utilized to describe the polymer elasticity of ELP (Elastin-like peptide) polypeptide (Fig. 2*B*, red dashed line). The force curve exhibited an average contour length of 35 nm (Fig. 2*D*), which is consistent with the theoretical value of ELP. Our result reveals the average unbinding force between MSU crystal and CLEC12A is determined as about 90 pN (Fig. 2*E*), while the CLEC12A-CTLD mutant rapidly decreases to 58 pN (Fig. S3), elucidating CLEC12A-CTLD has much higher affinity to MSU crystal at the atomic level and further verifying the critical role of “basic patch” involved in the binding of MSU crystals.

### CLEC12A receptors are expressed decreasingly upon MSU crystals sensing in macrophages

**Figure 3:**
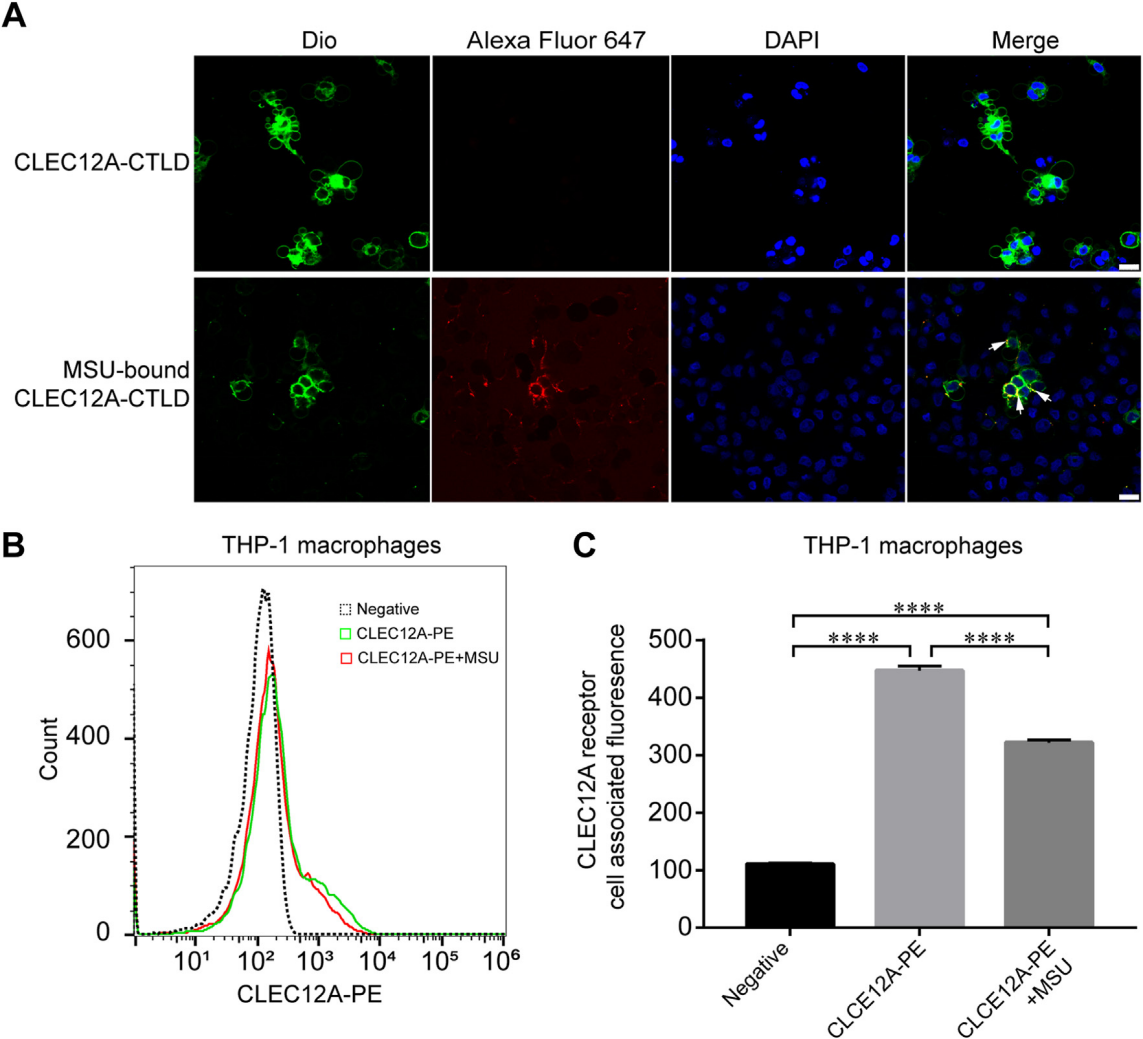
**Impact of MSU crystal treatment on CLEC12A receptor expression.***A*, representative images of binding of MSU-bound CLEC12A-CTLD-Alexa Fluor 647 to the surface of macrophages following MSU crystals treatment. Scale bar, 20 μm. *B*, flow cytometry analysis of THP-1 macrophages (unstained control), THP-1 macrophages strained with CLEC12A-PE (stained control), THP-1 macrophages treated with MSU crystals before stained with CLEC12A-PE. Histograms indicate the cell count of relative fluorescence intensity (n = 3). *C*, THP-1 macrophages were stained with CLEC12A-PE (stained control) or treated with MSU crystals and stained with CLEC12A-PE. Unstained THP-1 macrophages were used as control. Histograms indicate mean fluorescence intensity (MFI) staining of CLEC12A–positive cells in THP-1 macrophages (n = 3, means ± SEM, paired *t* test, ∗∗∗∗*p* < 0.0001). CTLD, C-type lectin-like domain.

Upon sensing MSU crystals, joint resident macrophages trigger inflammation through crystal phagocytosis and the release of IL-1β and other chemokines (32, 33, 34). C-type lectin-like receptors on macrophages extensively participate in the recognition of nanoparticles and crystals (35, 36). CLEC12A is expressed on macrophages and acts as inhibitory receptor in the modulation of MSU-induced inflammation (15, 36). In order to investigate the interaction between MSU crystals and THP-1 macrophages expressing CLEC12A, we used the labelled MSU crystals (bound with CLEC12A-CTLD Alexa Fluor 647) to incubate with THP-1 macrophages, then the macrophages were imaged by fluorescence microscopy. Over a 6-h treatment, the labeled MSU crystals adhered to the cell surface of THP-1 macrophages (Fig. 3*A*). In order to further detect the expression level of CLEC12A on the macrophage cell surface in response to MSU crystals stimuli, we also detected CLEC12A receptor expression level in MSU-treated THP-1 macrophages using flow cytometry. After treatment with MSU crystals, CLEC12A receptor slightly decreased as compared with the untreated control (Fig. 3, *B* and *C*). These results indicate CLEC12A receptors are involved in the internalization of MSU crystals following the binding to MSU crystals at the cell surface.

### CLEC12A clusters at “phagocytic vesicles” upon MSU crystals sensing

**Figure 4:**
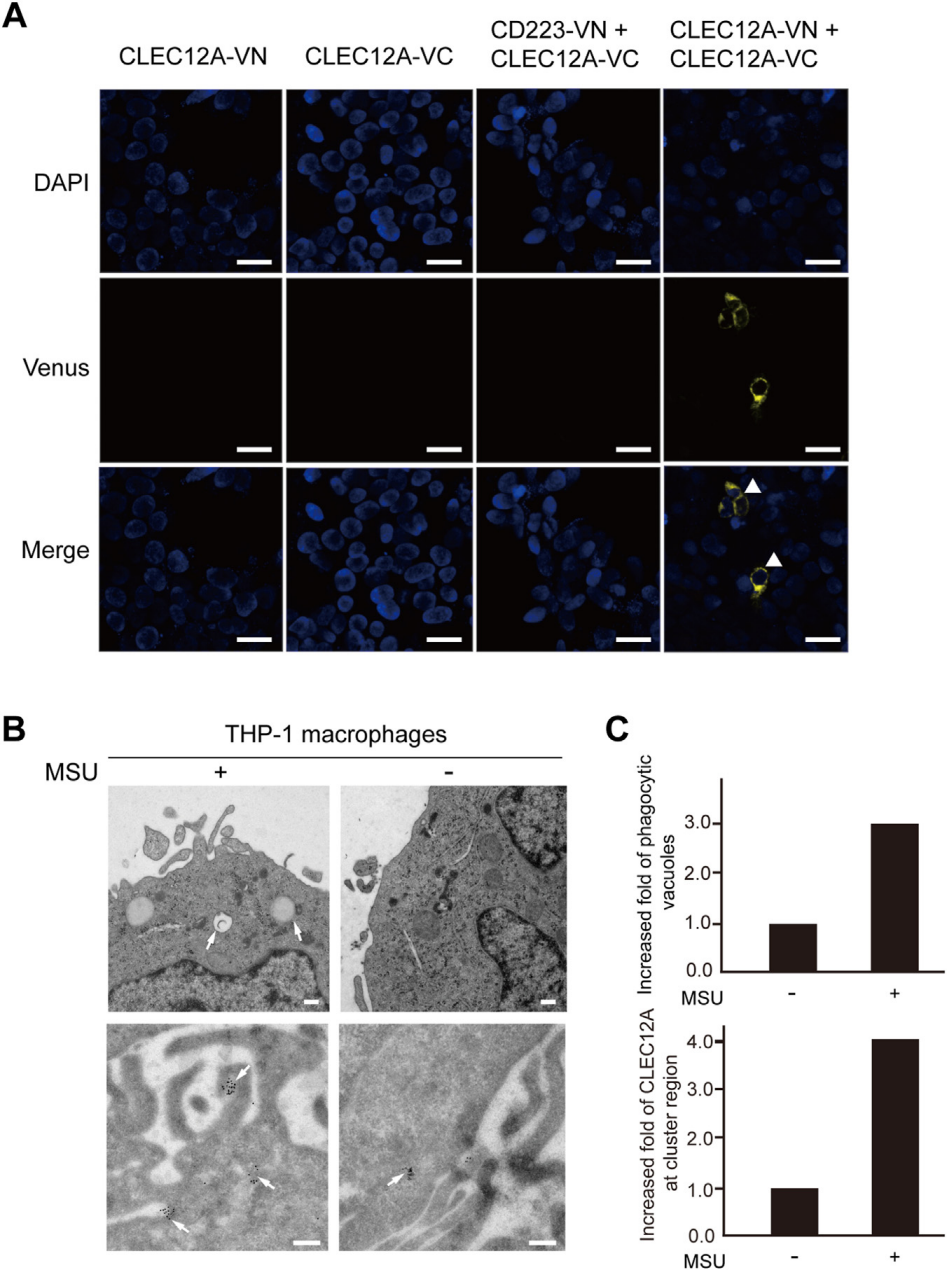
**CLEC12A distribution on the cell membrane and transmission electron microscopy of THP-1 macrophage stimulated with MSU crystals.***A*, confocal microscopy analysis and representative images of HEK293T cells transfected with CLEC12A-VN173, CLEC12A-VC155, CD223-VN 173+CLEC12A-VC155 or CLEC12A-VN173+CLEC12A-VC155, and stained with DAPI. Scale bar, 25 μm. *B*, conventional electron microscopy (*top*) and immune-electron microscopy (*bottom*) of MSU crystals treated or untreated THP-1 macrophage cells. Scale bar, 200 nm. *C*, increased fold of the phagocytic vesicles (*top*) and the CLEC12A molecules (*bottom*) at the cluster region after MSU crystals treatment.

To directly characterize and visualize the CLEC12A-mediated recognition of MSU crystals on the cell surface, we first determined the distribution and oligomerization of CLEC12A on the cell surface by bimolecular fluorescent complementation. And bimolecular fluorescent complementation showed that CLEC12A receptors preferentially oligomerize at the cell surface (Fig. 4*A*). Next, THP-1 macrophages treated with MSU crystals were observed under conventional electron microscopy and immune-electron microscopy. Electron micrographs showed THP-1 macrophage cells treated with MSU crystals exhibit swollen vesicular structures, and cavities of MSU crystals (Fig. 4*B*, top), which are similarly observed in synoviocytes (37, 38). Vesicles of different sizes induced by MSU crystals increased nearly 3-fold (6 per treated macrophage cell), in comparison to untreated macrophage cells (Fig. 4, *B* and *C*, top). Immunoelectron micrographs showed more CLEC12A receptors cluster at the phagocytic vesicles in MSU-treated macrophages than untreated macrophages (Fig. 4*B*, bottom). CLEC12A receptors increased about four-fold in MSU-treated macrophages than in untreated macrophages (Fig. 4, *B* and *C*, bottom). Electron microscopic observation revealed CLEC12A receptors oligomerize at the “phagocytic synapse” structure that is also found in CLEC9A and CLEC7A receptors (27, 39, 40, 41). Also, a recent finding also shows that CLEC12A oligomerizes *via* cysteine residues in the stalk region (42), which is in agreement with our findings. Collectively, these results indicate that CLEC12A receptors cluster or oligomerize in a manner of “phagocytic vesicles” at the cell surface during the recognition and internalization of MSU crystals by macrophages.

## Discussion

CLEC12A is a myeloid inhibitory receptor playing an important role in negatively regulating MSU-induced inflammation (30). Similar to other members of C-type lectin receptors, CLEC12A harbors a canonical C-type lectin domain that is responsible for ligand binding. Although the C-type lectin domains adopt a conserved conformation, their selectivity and specificity for ligand-binding are different. Here, we reported the high-resolution crystal structure of hCLEC12A-CTLD and the mutant assay also proves that CLEC12A “basic patch” plays a critical role in the recognition of MSU crystals. For the first time, we determined the single-molecule force between CLEC12A and MSU crystals by AFM-SMFS. Around 90 pN unbinding force between CLEC12A and MSU crystals is directly measured, indicating CLEC12A has a strong interaction with MSU crystals compared with the receptor binding domain from SARS-CoV-2 with ACE2 (43). In particular, this interaction force contributes to a better understanding of the molecular recognition event between CLEC12A and MSU crystals, despite the current knowledge of the binding characteristics between them is limited. The structural diversity of CTLDs in CLRs contributes to recognizing different ligands, which is mainly dependent on the specific long-loop regions. The crystal structure of CLEC12A-CTLD reveals a distinct “basic patch” feature that is required for the recognition of MSU crystals. Similarly, this feature is also found in one of the dectin-1 cluster members, LOX-1, which harbors a basic spine structure region composed of positively charged residues arginines, which is essential for LOX-1 binding (44).

Moreover, we found that CLEC12A receptors tend to cluster or oligomerize on the cell membrane, which is consistent with recent reports that CLEC12A stalk cysteines are necessary for CLEC12A oligomerization (42). Flow cytometry in combination with electron microscopic observations demonstrate that upon stimulated by MSU crystals, CLEC12A receptors decreased on the surface of THP-1 macrophages and CLEC12A receptors oligomerize at the “phagocytic synapse” in response to MSU crystals danger signals, which suggests that CLEC12A is involved in the internalization of MSU crystals. Although MSU crystals could trigger the activation of the NLRP3 inflammasome and a strong inflammatory response *via* TLRs (30), CLEC12A plays inhibitory roles in negatively downregulating the inflammatory response through not only the ITIM motif but also the internalization of MSU crystals. Gout is caused by the deposition of MSU crystals in articular and non-articular structures, exhibiting gout flares with intense pain. Whether gout flares can be alleviated by activating CLEC12A inhibitory signals or promoting the phagocytosis of MSU crystals by CLEC12A needs to be further investigated. Taken together, our observations provide the first demonstration of the interaction between CLEC12A and MSU crystals from not only molecular but also cellular perspectives, giving an insightful example of the interplay between cell surface receptors and endogenous crystals.

## Experimental procedures

### Preparation of MSU crystals

MSU crystals were prepared as previously described (45). 1 g of uric acid (Sigma-Aldrich) was dissolved in 200 ml boiled distilled water containing 0.03 M NaOH. HCl solution was added to adjust the pH to 7.4, and the solution was stirred slowly and continuously at room temperature. 12 h later, the crystals were harvested, autoclaved, and suspended in PBS buffer (pH 7.4).

### Protein expression and purification

For crystallization, the CTLD (Pro132-Val255) of the human CLEC12A gene was constructed into pFastBac1 vector and fused with an N-terminal IL-2 signal peptide and a C-terminal 6× His tag. Recombinant baculoviruses were packaged in Sf9 cells following the baculovirus expression protocol (Invitrogen). Hi5 cells were used for protein expression using ESF921 medium (Expression Systems). The supernatant was collected after 72 h of infection and dialyzed against 20 mM Tris, 150 mM NaCl, pH 8.0, and then applied to nickel-nitrilotriacetic acid beads followed by Superdex 200 increase column purification. The fractions corresponding to the hCLEC12A-CTLD protein were pooled and concentrated to 16 mg/ml for crystallization. For mutagenesis studies, the hCLEC12A-CTLD wild (Pro132-Val255) gene was constructed into pcDNA3.4 vector and fused with an N-terminal IL-2 signal peptide and a C-terminal 6× His tag. The hCLEC12A-CTLD mutant (R185A/R201A/R204A/R232A) and single-site mutations were generated using KOD-Plus-Mutagenesis Kit (TOYOBO). HEK293F cells were transiently transfected with pcDNA3.4-hCLEC12A-CTLD-mutant or single-site mutation plasmids *via* polyethyleneimine. The supernatant was collected after transfection for 5 days, and the subsequent purification procedure was the same as described earlier.

### Crystallization and structure determination

Crystallization of the hCLEC12A-CTLD protein from Hi5 cell was carried out with the sitting-drop vapor-diffusion method by mixing equal volumes (1 μl) of protein (16 mg/ml) and reservoir solution from JCSG core kits (Qiagen) at 16 °C. The crystal for the hCLEC12A-CTLD protein was grown in a solution containing 20% (w/v) PEG 3350, 0.2 M Potassium sulfate. Diffraction data were collected at the BL19U beamline of the National Facility for Protein Science Shanghai (NFPS) at the Shanghai Synchrotron Radiation Facility (SSRF) and processed by the XDS program (46). The diffraction data statistics are summarized in Table S1. The crystal structure of hCLEC12A-CTLD was solved by molecular replacement using the structure of the CTLD domain of hCLEC9A (Protein Data Bank ID:3VPP) as a search model. Molecular replacement, model building, and refinement were performed using the PHENIX suite (47) and Coot (48). The refinement statistics are summarized in Table S1.

### Fluorescence microscopy

For the binding of protein to MSU crystals, the wild-type of hCLEC12A-CTLD and the mutant of hCLEC12A-CTLD proteins were labeled with Alexa Fluor 647 NHS Ester (Molecular Probes) according to the manufacturer’s protocol. Briefly, the purified protein (2 mg/ml) was added with a 10-fold molar excess of Alexa Fluor 647 NHS Ester in PBS buffer (pH7.4), incubated for 2 h at room temperature. The labelled protein was desalted by Zebra Spin Desalting Columns (Thermo Fisher Scientific), and the fluorescent dye/protein ratio was determined by UV spectroscopy. Around 100 μg of MSU crystals were incubated in Alexa Fluor 647-labelled wild-type hCLEC12A-CTLD protein (100 μg/ml) or the mutant hCLEC12A-CTLD protein (100 μg/ml) for 1 h, respectively. MSU crystals were mounted under cover slips on glass slides after the crystals were washed three times with PBS buffer (pH7.4). Images were acquired with 20× magnification and observed with a TCS SP8-MaiTai M confocal microscopy (Leica).

For the binding of MSU crystals by macrophages, THP-1 cells were differentiated into macrophage-like cells by stimulation with 0.1 ng/μl phorbol 12-myristate 13-acetate (PMA) for 24 h, followed by another 24-h culture in normal medium. CLEC12A-CTLD protein was labelled with Alexa Fluor 647 as described earlier. THP-1 macrophages were treated with MSU crystals bound with Alexa Fluor 647 NHS Ester labeled CLEC12A-CTLD at 37 °C. After incubation for 6 h, THP-1 macrophages were washed in PBS for 3 times and stained with DIO and DAPI. Confocal imaging was performed using TCS SP8-MaiTai M confocal microscopy (Leica).

For the bimolecular fluorescent complementation assay, HEK293T cells were maintained and cultured in DMEM medium with 10% FBS. HEK293T cells were transfected with pcDNA3.4- CLEC12A-VN173 or pcDNA3.4- CLEC12A-VC155, and co-transfected with pcDNA3.4-CD223-VN173 and pcDNA3.4-CLEC12A-VC155, or pcDNA3.4-CLEC12A-VN173 and pcDNA3.4-CLEC12A-VC155. After post-transfection of 24 h, HEK293T cells were observed under TCS SP8-MaiTai M confocal microscopy (Leica).

### AFM-SMFS

Atomic force microscope (Nanowizard4, JPK) was used to perform the AFM-SMFS experiment. Firstly, the target protein hCLEC12A-CTLD or CTLD mutant with a N-terminal Asn-Gly-Leu (NGL) amino acids sequence was prepared as above described and site-specifically immobilized onto the Gly-Leu-ELP (Elastin-like peptide)-coated AFM tip *via* an OaAEP1 ligase-mediated ligation (49). To prepare the substrate, the surface of a polystyrene culture dish is initially treated with a coating of poly-l-lysine. Subsequently, MSU crystals are carefully positioned onto the treated dish surface in order to facilitate AFM experiments. Secondly, approaching the CTLD-coated AFM tip to the MSU crystals, and CTLD bind to the crystal upon contacting. Finally, the tip was withdrawed and CTLD was unbound, leading to a detachment force peak if a direct interaction was present. Sequentially, the tip was moved to the control position (buffer without MSU crystals) and repeated this cycle/measurement thousands of times, leading to a statistical unbinding force value. Accurate spring constant from functionalized MLCT-Bio-DC cantilever (Bruker) was acquired by a thermally induced fluctuation method. Peptide linker, C-ELP20-GL, was used to functionalize AFM tips. Typically, the tip contacted the crystal for 400 ms under an indentation force of 450 pN to ensure a site-specifically interaction between CLEC12A and the crystal. Then, the tip was moved up vertically at a constant velocity of 1000 nm/s, and the bound complex ruptured. The data were first filtered by JPK data processing and then analyzed by Igor Pro 6.12, only typical single molecule saw-tooth-like curves were chosen. The worm-like chain model (Equation 1) was used to fit curves with a persistence length of ∼0.4 nm.

(1) $$F\left(x\right)=\frac{k_{B}T}{p}\left[\frac{1}{4}{\left(1-\frac{x}{L_{c}}\right)}^{-2}-\;\frac{1}{4}+\frac{x}{L_{c}}\right]$$

where *F(x)* is the force applied to the polymer (polypeptide chain) under a polymer extension *x*. *P* is the persistence length of the polymer. *L*_*c*_ is the contour length. *k*_*B*_ is the Boltzmann constant, and *T* is the temperature in kelvin.

Gaussian function (Equation 2) was used to fit the histogram

(2) $$f\left(x\right)=W_{0}+W_{1}*e^{{-\left(\frac{x-w_{2}}{w_{3}}\right)}^{2}}$$

where *W*_*0*_, *W*_*1*_, *W*_*2*_ and *W*_*3*_ are arbitrary real constants (*W*_*3*_≠0).

### Flow cytometry

The human monocytic cell line THP-1 was cultured in RPMI1640 medium containing 10% FBS and 1% Penicillin/Streptomycin. Cells were differentiated into macrophage-like cells by stimulation with 0.1 ng/μl phorbol 12-myristate 13-acetate (PMA) for 24 h, followed by another 24-h culture in normal medium. For cell-surface binding assay, THP-1 macrophages were incubated with 100 μg of MSU microcrystals (ultrasonic treatment for 1 h) per 10,000 cells for 20 min. For negative control, THP-1 macrophages were incubated without MSU crystals. After incubation, cells were collected (THP-1 macrophages were detached by 5-min incubation in 2 mM EDTA at 37 °C) and washed twice in PBS buffer. THP-1 macrophages were then stained with PE-anti-human CLEC12A mAb (BioLegend) followed by washing with PBS buffer for three times. Flow cytometry data was collected in an Attune N x T cytometer (Thermo Fisher Scientific). Data analysis was performed using FlowJo software (Tree Star).

### Electron microscopy

For conventional-electron microscopy, the THP-1 macrophages treated with/without MSU crystals for 3 h were fixed with 2.5% glutaraldehyde, post-fixed for 1 h with 1% osmium tetroxide, and dehydrated in a graded series of alcohol. The samples were embedded in an epoxy resin and polymerized at 60 °C for 24 h. Sections were cut 60 to 70 nm thick, and stained with 4% uranyl acetate and lead citrate. The cells were then observed under a Talos L120C transmission electron microscope (Thermo Fisher Scientific).

For Immuno-electron microscopy, the THP-1 macrophages treated with/without MSU crystals for 3 h were fixed with 4% paraformaldehyde and 0.1% glutaraldehyde. After 1 h, the cells were rinsed in PBS buffer for four times and embedded with 12% gelatin. The gelatin slices containing cells were sectioned into cubic blocks. For cryoprotection, blocks were infiltrated overnight with 2.3 M sucrose at 4 °C, mounted on aluminum pins, and frozen in liquid nitrogen. Cryo-ultrathin-sectioning was carried out at 120 °C. Sections were picked up with 2.3 M sucrose and transferred to carbon-coated formvar nickel grids. For Immunogold labeling of ultrathin sections, grids were labeled with mouse anti-human CLEC12A polyclonal antibody (Solarbio) and goat anti-mouse IgG H&L secondary antibody conjugated with 10 nm-gold particles (Abcam). Grids were stained with 2% methyl cellulose (contain 0.2% uranyl acetate) and air-dried at room temperature. Sections were observed and imaged with a Talos L120C transmission electron microscope (Thermo Fisher Scientific).

## Data availability

The structure of the CTLD domain of human CLEC12A has been deposited in the RCSB Protein Data Bank under the accession codes 8JAH.

## Supporting information

This article contains supporting information.

## Conflict of interest

All authors declare that they have no competing interests.

## Figures and Tables

**Supporting Figure S3:**