# Supplementary material for: Mechanistic insights into the C-type lectin receptor CLEC12A-mediated immune recognition of monosodium urate crystal
Source: J Biol Chem. 2024 Feb 16;300(3):105765. doi: 10.1016/j.jbc.2024.105765 (PMC10959670; doi:10.1016/j.jbc.2024.105765)
Supplement: Supporting Information [file mmc1.docx]

**Mechanistic insights into the C-type lectin receptor CLEC12A-mediated immune recognition of monosodium urate crystal**

Hua Tang^1, 2^*†, Yuelong Xiao^3^*, Lei Qian^1^, Zibin Wang^1^, Ming Lu^5^, Nan Yao^1^, Ting Zhou^1^, Fang Tian^3^, Longxing Cao^5^, Peng Zheng^3^†, Xianchi Dong^1, 2, 4^†

^1^State Key Laboratory of Pharmaceutical Biotechnology, Department of Biochemistry, School of Life Sciences, Nanjing University; Nanjing, 210023, China.

^2^Engineering Research Center of Protein and Peptide Medicine, Ministry of Education; Nanjing, 210023, China.

^3^State Key Laboratory of Coordination Chemistry, Chemistry and Biomedicine Innovation Center (ChemBIC), School of Chemistry and Chemical Engineering, Nanjing University, Nanjing, Jiangsu, 210023, China.

^4^Institute of Artificial Intelligence Biomedicine, Nanjing University; Nanjing, 210023, China.

^5^Westlake laboratory, Westlake University, Hangzhou, Zhejiang, 310024, China.

†Corresponding authors. E-mail: Xianchi Dong, xianchidong@nju.edu.cn; Peng Zheng, pengz@nju.edu.cn; Hua Tang, tanghua@nju.edu.cn.

**Supplemental Information**

**Supplemental Methods**

**Protein-MSU crystal pulldown**

Purified CLEC12A-CTLD WT or mutations (R185A, R201A, R204A, R232A, and quadruple mutant) were respectively incubated with MSU crystals for 60 min at 4 °C. After incubation, MSU crystals were pelleted by centrifugation and washed in PBS buffer (20 mM Na_3_PO_4_, 150 mM NaCl, pH 7.4) for 3 times. After spin-down and washing, MSU crystals or protein inputs were boiled with beta-mercaptoethanol. Both protein inputs and MSU crystals pellet outputs were loaded onto SDS-PAGE.

**CD spectrometry**

Far-ultraviolet circular dichroism measurements were carried out with a Chirascan-Plus-V100 instrument equipped with a temperature-controlled multi-cell holder. Wavelength scans were measured from 280 to 190 nm at 25 °C. Temperature melts monitored the dichroism signal at 222 nm in steps of 2 °C min–1 with 30 s of equilibration time. Wavelength scans and temperature melts were performed using 0.5 mg ml^–^1 protein in PBS buffer (20 mM Na_3_PO_4_, 150 mM NaCl, pH 7.4) with a 1 mm path-length cuvette. Melting temperatures were determined by fitting the data with a sigmoid curve equation.

**Supplemental Figure Legends**

**Supplemental Figure S1. Protein-MSU crystal interaction pulldown. (A)** CTLD WT and mutated proteins are pulled down with MSU crystals, and Mutant (Combo, * *) exhibits apparent reduction of binding to MSU crystals. (B) Calculation of relative grey intensity ratios between pulldown outputs. Mutant-to-WT ratio is 59.9 %, showing the weakest binding affinity in all different mutations.

**Supplemental Figure S2. CD spectra for the wild-type and mutant** **CLEC12A-CTLD. (A)** The far-UV CD spectra for the wild type (line) and mutant protein (dashed line) are similar at 25 °C. **(B)** Thermal denaturation of CLEC12A-CTLD WT and CLEC12A-CTLD mutant from 25 °C to 95 °C. Molar ellipticity at 222 nm was measured, and the values were used to calculate a mole fraction of denatured molecules for each temperature. Midpoints of the transitions (WT, 63.1 °C; Mutant, 64.5 °C) are determined.

**Supplemental Figure S3. Measurement of the binding strength between hCLEC12A-CTLD mutant and MSU crystal by AFM-SMFS. (A)** Force-extension curves of the unbinding between CLEC12A-CTLD mutant and MSU crystal, showing a force peak with a contour length of ~42 nm and a force of ~58 pN. **(B)** The histogram of contour length (Lc) of the unbinding force peak showed an average length of 42 nm. **(C)** The histogram of unbinding force between CLEC12A-CTLD mutant and MSU crystal showed an average force of 58 pN.
