# Supplementary material for: Mechanistic insights into the C-type lectin receptor CLEC12A-mediated immune recognition of monosodium urate crystal
Source: J Biol Chem. 2024 Feb 16;300(3):105765. doi: 10.1016/j.jbc.2024.105765 (PMC10959670; doi:10.1016/j.jbc.2024.105765)
Supplement: Supporting Table S1 [file mmc2.docx]

Table 1. Data collection and refinement statistics.

| Wavelength (Å)  Resolution (Å) | 0.97853  42.16 - 2.581 (2.671 - 2.581) |
| --- | --- |
| Space group | P 61 2 2 |
| Unit cell  a, b, c (Å)  α, β, γ (°) | 50.012 50.012 367.627  90 90 120 |
| Redundancy | 13.4 (13.7) |
| Completeness (%) | 92.38 (82.80) |
| Mean I/sigma(I) | 13.357 (1.462) |
| Rmerge | 0.206 (0.953) |
| Rmeasure | 0.206 (0.953) |
| No. reflections used for Rwork | 8836 (751) |
| No. reflections used for Rfree | 419 (34) |
| Rwork | 0.2183 (0.2449) |
| Rfree | 0.2758 (0.2710) |
| Number of non-hydrogen atoms | 2031 |
| Protein residues | 244 |
| R.m.s. deviations  Bond lengths (Å)  Bond angles (°) | 0.0081  0.97 |
| Ramachandran favored (%) | 94.17 |
| Ramachandran allowed (%) | 5.83 |
| Ramachandran outliers (%) | 0.00 |
| Rotamer outliers (%) | 0.90 |
| Clashscore | 8.31 |
| Average B-factor | 41.60 |

Statistics for the highest-resolution shell are shown in parentheses.
